# Oral immunotherapy be heated ovomuciod-reduced egg white in a Balb/C mouse model

**DOI:** 10.1186/2045-7022-1-S1-O35

**Published:** 2011-08-12

**Authors:** Rodrigo Jiménez-Saiz, Chengbo Yang, Rosina López-Fandiño, Yoshionori Mine

**Affiliations:** 1 Instituto de Investigación en Ciencias de la Alimentación (CIAL) CSIC-UAM, Madrid, Spain; 2 University of Guelph, Food Science, Guelph, Canada

## Background

Food allergies are a problematic health concern in many developed countries. Oral immunotherapy (OIT) is one of the most promising therapeutic approaches for treating food allergy. The treatment with heated ovomucoid-reduced egg white (OM‾) is especially notable and its effectiveness as OIT in egg allergic patients has been reported: 24 (44%) egg allergic patients became tolerant after two months of OIT with OM‾. However a better understanding of molecular mechanisms underlying the OIT is not well established.

## Methods

OIT using OM‾ was carried out with an egg-allergy Balb/c mouse. The mice were sensitized by intact EW and then de-sensitized by OM‾ by means of oral ingestion. Histamine levels and specific IgE, IgG and IgG2a against EW allergens in sera were measured by ELISA. Splenocytes were cultured in the presence of EW. IFN-γ (Th1), IL-4 (Th2), IL-10 and TGF-β (T-reg) were assessed in cell cultured supernatants. Fecal samples were collected weekly and processed for analyzing EW-specific IgA.

## Results

treated mice showed significantly lower histamine release and EW-specific IgE activity compared to positive group. A significant increase of ovomucoid and ovalbumin specific IgG2a was found in treated mice sera. The IL-4 was significantly suppressed and enhancement of IFN-γ and IL-10 was observed in the treatment group, however there was no difference in TGBβ concentrations. Interestingly the treated group showed higher secretions of EW- specific IgA in fecal samples than the positive and negative group.

## Conclusions

OIT using OM‾ led to oral desensitization by inducing an increase of Th1/Th2 ratio. The high up-regulation of IL-10 in treated mice suggests that regulatory T-cells played an important role in oral desensitization. The high activity of ovomucoid and ovalbumin specific IgG2a in sera corroborate the enhancement of Th1 response. The increase of secreted EW specific-IgA in the treated group fecal samples might have contributed to mucosal immunity to the suppression of egg allergic responses using OM‾.

